# Effects of soluble guanylate cyclase stimulator on renal function in ZSF-1 model of diabetic nephropathy

**DOI:** 10.1371/journal.pone.0261000

**Published:** 2022-01-27

**Authors:** Lufei Hu, Yinhong Chen, Xiaoyan Zhou, Maarten Hoek, Jason Cox, Ken Lin, Yang Liu, Wendy Blumenschein, Jeff Grein, Gayathri Swaminath

**Affiliations:** 1 Department of Cardiometabolic Diseases, Merck & Co., Inc., Kenilworth, NJ, United States of America; 2 Quantitative Biosciences, Merck & Co., Inc., Kenilworth, NJ, United States of America; 3 Biology Department, Maze Therapeutics, San Francisco, CA, United States of America; 4 Chemistry, Merck & Co., Inc., Kenilworth, NJ, United States of America; 5 Discovery Chemistry, Kinnate Biopharma, San Diego, CA, United States of America; 6 Pharmacokinetics, Pharmacodynamics & Drug Metabolism, Merck & Co., Inc., Kenilworth, NJ, United States of America; 7 Drug Metabolism and Pharmacokinetics, BridgeBio, Palo Alto, CA, United States of America; 8 Department of Molecular Discovery Profiling and Expression, Merck & Co. Inc., Kenilworth, NJ, United States of America; Max Delbruck Centrum fur Molekulare Medizin Berlin Buch, GERMANY

## Abstract

**Background:**

Diabetic nephropathy is associated with endothelial dysfunction and oxidative stress, in which the nitric oxide-soluble guanylate cyclase-cyclic guanosine monophosphate (NO-sGC-cGMP) signaling pathway is impaired. We hypothesize that sGC stimulator Compound 1 can enhance NO signaling, reduce proteinuria in a diabetic nephropathy preclinical model with diminished NO bioavailability and increased oxidized sGC. Therefore, we evaluated the effect of sGC stimulator Compound 1 on the renal effect in obese ZSF1 (ZSF1 OB) rats.

**Materials and methods:**

The sGC stimulator Compound 1, the standard of care agent Enalapril, and a combination of Compound 1 and Enalapril were administered chronically to obese ZSF1 rats for 6 months. Mean arterial pressure, heart rate, creatinine clearance for glomerular filtration rate (eGFR), urinary protein excretion to creatinine ratio (UPCR), and urinary albumin excretion ratio (UACR) were determined during the study. The histopathology of glomerular and interstitial lesions was assessed at the completion of the study.

**Results:**

While both Compound 1 and Enalapril significantly reduced blood pressure, the combination of Compound 1 and Enalapril normalized blood pressure levels. Compound 1 improved eGFR and reduced UPCR and UACR. A combination of Enalapril and Compound 1 resulted in a marked reduction in UPCR and UACR and improved GFR.

**Conclusion:**

The sGC stimulator Compound 1 as a monotherapy slowed renal disease progression, and a combination of the sGC stimulator with Enalapril provided greater renal protection in a rodent model of diabetic nephropathy.

## Introduction

Chronic Kidney Disease (CKD) is a huge unmet need requiring novel efficacious treatments to address disease progression. The nitric oxide-soluble guanylate cyclase-cyclic guanosine monophosphate (NO-sGC-cGMP) signaling cascade pathway plays a critical role in regulating renal function. Cyclic guanosine monophosphate (cGMP) vasodilates renal vasculature and directly influences renal blood flow, renin secretion, glomerular function, and tubular exchange processes [1, 2]. Impairment of NO-sGC-cGMP signaling results in severe kidney disease such as CKD [3].

Diabetic nephropathy (DN) is the most common cause of end-stage renal disease (ESRD) in Western societies and is characterized by a progressive decline in renal function accompanied by mesangial expansion, glomerular basement membrane thickening, and tubulointerstitial damage [4, 5]. Endothelial dysfunction is the leading cause for the progression of DN. The NO-sGC-cGMP signaling pathway plays a central role in mediating a variety of physiological responses, including smooth muscle relaxation, metabolism, mitochondrial biogenesis, and vascular and platelet homeostasis [6–9]. Endogenous nitric oxide (NO) is generated by endothelial NO synthesis (eNOS) from L-arginine. When released from the endothelium, NO binds to the ferrous (Fe^2+^) heme of the “native” soluble guanylate cyclase (sGC) and increases sGC activity and the formation of cGMP from compromised guanosine triphosphate (GTP) [10, 11]. Multiple diseases and conditions such as hypertension, heart failure, CKD, and metabolic syndrome are associated with reduced NO bioavailability due to endothelial dysfunction [12–20].

The sGC stimulator binds to an allosteric site of sGC and potentiates NO-sGC signaling [21, 22]. sGC stimulators are expected to be beneficial in disease states in which NO bioavailability is impaired or the sGC enzyme is oxidized. As kidney disease progresses, there is severe endothelial dysfunction with low NO production and sGC oxidation. sGC stimulators alone can enhance the activity of endogenous NO and act synergistically in the presence of NO to improve kidney function. Because the predominant defect—NO bioavailability or an sGC oxidative state—can vary in different diseases, different tissues or cells, or even in different stages of the disease, an sGC stimulator could be more advantageous over the other sGC class. Indeed, sGC compounds have demonstrated organ-protective effects in a variety of preclinical animal models with cardiovascular or renal dysfunction [23–32]. Given that DN is associated with endothelial dysfunction and increased oxidative stress due to low levels of NO and an oxidative state of sGC, treatment strategies aiming to vasodilate renal vasculature via the sGC stimulator may have beneficial effects for the treatment of progressive kidney disease. We hypothesize that the sGC stimulator Compound 1 is protective against DN at stages in which NO bioavailability is low or diminished. The present study was designed to assess systemic hemodynamic renal function and histology in ZSF1 rats (Lepfa/Leprcp) [a hybrid F1 of female Zucker diabetic fatty rat (Lepfa) and male spontaneously hypertensive heart failure rat (Leprcp) with different leptin receptor mutation] because these rats exhibit many of the traits of human diabetic nephropathy, including proteinuria, renal lesions, hyperglycemia, dyslipidemia, mild hypertension, oxidative stress, and obesity [33–36]. Acute and chronic studies were performed to assess renal function. The acute study investigated the effect of the sGC stimulator Compound 1 on renal blood flow and blood pressure (BP). The chronic study investigated the protective role in renal function of the vasodilator effect of sGC combined with the standard of care drug Enalapril, an angiotensin-converting enzyme inhibitor.

## Materials and methods

### Animals and reagents

#### Cell-based sGC functional assay (CASA assay)

The in vitro activity of compound 1 was assessed in CASA assay using CHO-K1/sGC stable cell line. In brief, A CHO-K1 cell line stably expressing sGC was generated. CHO-K1 cells were transfected with plasmids plREShyg-hsGCC 1 and plRESneo-hsGCB1 simultaneously using FUGENE reagent. Clones that stably express both subunits 50 were selected with hygromycin and neomycin. Clone #7 was chosen for the assay and was designated CHO-K1/sGC. CHO-K1 /sGC cells were maintained in F-K12 medium containing 10% heat-inactivated Fetal Bovine Serum (FBS), 100 ug/mL penicillin/streptomycin, 0.5 mg/mL hygromycin and 0.25 mg/mL G418. On the day of the assay, cells were harvested in EBSS Assay Buffer (EAB) containing 5 mM MgCl, 10 mM HEPES (4-(2-hydroxyet hyppiperazine-1-ethanesulfonic acid) and 0.05% BSA (bovine serum albumin) and cell density was adjusted to 2x10/mL with EAB. IBMX (3-isobutyl-1-methylxanthin, 0.5 mM) was added to inhibit degradation of cGMP. Compound 1 was diluted from DMSO stock solutions and added to the assay at a final DMSO concentration of 1%. Cells were incubated with compound 1 in the presence and absence of 10 uM of 1H-(12.4) oxadiazolo(4.3-a) quinoxalin-1-one (ODQ) for 1 hr at37° C. At the end of the incubation period, the reaction was terminated, and the cells were lysed. The level of intracellular cGMP was determined using an HTRF-based assay kit (CisBio. 62GM2PEC).

For the acute study, Sprague Dawley (CD) rats were obtained from Charles River Laboratories (CRL, Kingston, NY) in an anesthetized condition. Male ZSF1 obese (ZSF1 OB) rats (ZSF1LeprfaLeprcp/Crl) were obtained from CRL around 15 weeks of age. For the chronic study, telemetry transmitters (HD-s10, DSI, Data Sciences International, St. Paul, MN) were implanted under ketamine (80mg/kg) /xylazine (8mg/kg) i.p. To extend the duration of anesthesia plane, ketamine boost was given (not exceeding 50% of the original doses of ketamine) as needed during procedure. The detail procedure of telemetry catheter implantation was followed as per DSI manufactory manual. Briefly, A 4–6 cm midline abdominal incision was made. The transmitter was inserted into abdominal aorta. The implanted telemetry system allows for continuous monitoring of BP and heart rate (HR). Rats were acclimated to metabolism cages for 1 week prior to the start of urine collection. All procedures utilizing experimental animals were conducted in accordance with the Guide for the Care and Use of Laboratory Animals, and experimental procedures were reviewed and approved by the Institutional Animal Care and Use Committee (IACUC) at the research laboratories of Merck & Co., Inc., Kenilworth, NJ, USA.

Compound 1 (soluble guanylate cyclase activators, US 9,365,574 B2) was synthesized by Merck & Co., Inc., Kenilworth, NJ, USA. Both Enalaprilat (catalog number 1235274) and Enalapril (catalog number E6888) were purchased from Sigma-Aldrich (St. Louis, MO). For the chronic study, the medicated diets (either Compound 1, Enalapril or a combination mixed in Purina Rodent Chow 5053 (LabDiet, St. Louis, MO) were prepared by Research Diets, Inc. (New Brunswick, NJ). The compounds were administered by in-feed dosing; the compound in feed concentration was determined by body weight and daily food intake based on historical data of daily food intake (34 g/day). The treatment was started with 22-week-old ZSF1 OB rats and continued for 6 months. Animals were housed in a temperature- and humidity-controlled facility with a 12:12-hour dark-light cycle with ad libitum access to food (Purina Rodent Chow 5053) and water.

### Experiment design

For the pilot study, the acute study male CD rat average weight was 449 ± 10 g at 12 weeks old. Three treatments were administrated intravenously over 30 minutes: 1) vehicle control [Dimethyl Sulfoxide/ polyethylene glycol 400/H2O, 10%/60%/30% (v/v/v)]; 2) Compound 1 treatment at a low dose at 0.05 mg/kg; and 3) Compound 1 treatment at a higher dose at 0.1 mg/kg. The effect of Compound 1 alone or combined with Enalaprilat was evaluated on the disease-relevant model. Forty male ZSF1 obese rats with an average body weight of 618 ± 9 g at 28 weeks old were given 4 treatments with n = 10 per group: 1) vehicle control [Dimethyl Sulfoxide/ polyethylene glycol 400/H2O, 10%/60%/30% (v/v/v)]; 2) Compound 1 dosed at 0.1 mg/kg over 30 minutes intravenously (IV); 3) Enalaprilat 3 mg/kg; 4) combination of Compound 1, 0.1 mg/kg, and Enalaprilat 3 mg/kg over 30 minutes IV. Under brief anesthesia with Inactin (T133-1G, Sigma-Aldrich; 100 mg/kg IP), a trachea cannulation was performed using polyethylene (PE-200) tubing, and the carotid artery was cannulated with PE-50 tubing and connected to an ADInstruments PowerLab 16/35 (Colorado Springs, CO) for continuous recording of BP and HR. The jugular vein was cannulated with PE-50 tubing for continuous IV drug administration using the Harvard infusion pump PHD2000 (Cambridge, MA). A Stryker T/Pump (Kalamazoo, MI) water bath and heating pad was used to maintain rat body temperature around 37°C. An ultrasonic transit time (1RB, Transonic Systems; Ithaca, NY) flow probe was placed around the left renal artery for measurement of renal blood flow (RBF) by a flowmeter Transonic system TS420 (Ithaca, NY) to record beat-to-beat RBF. The RBF was allowed to stabilize for at least 30 min followed by 30 min of baseline measurement recorded before drug administration. All compounds were infused over a 30-minute period and infusion was stopped and recorded for another 30 minutes. Terminal blood samples were taken to measure drug exposure level. The animals were euthanized with an IV overdose of Inactin.

For the 6-month chronic study, 44 male ZSF1 OB rats (22 weeks of age), weighted 596 ± 4 g were randomly assigned for 26 weeks into 4 groups based on their average baseline urinary protein excretion (UPE), mean blood pressure (MBP), and body weight to receive either vehicle control (n = 11), Enalapril (3 mg/kg/day in chow, n = 9/group), or Compound 1 at 1 to 3 mg/kg/day (n = 12), or a combination of Compound 1 at 1 mg/kg/day with Enalapril (3 mg/kg/day) (n = 12/group). Heart rate, systolic pressure, diastolic pressure and mean BP were collected for 60 seconds every 5 minutes via DSI Dataquest system version 4.1 (Data Sciences International, St. Paul, MN) for 5 days every other week. Body weight and food and water intake were recorded biweekly. It is known that hemodynamics response could indirectly affect renal function. The study was intended to see if sGC stimulator alone would have benefits compared to standard of care drugs such as enalapril. The hemodynamic response was maintained similar to enalapril to investigate direct renal effects and not confound with blood pressure effects. Compound 1 was initiated at low dose of 1mg and increased to 3mg/kg to have sustained blood pressure comparable to enalapril effect on these rats. Animals were individually housed during scheduled telemetry recording and metabolism urine collection to allow for collection of individual animal data and samples. Urine was collected biweekly over a 24-hour period for measurement of protein and albumin. Rats were acclimated to metabolism cages for at least 1 week prior to baseline urine collections and remained in these cages to allow for biweekly collection of urine. Baseline measurements were collected 1 week prior to the start of treatment at 21 weeks of age. Urine was collected at room temperature and urine volume was recorded for each rat; blood samples were obtained right after 24-hour urine collection by jugular vein puncture. Blood samples were collected at baseline, biweekly for the first 2 months and monthly afterward, over 26 weeks of treatment and at termination of the study for pharmacokinetic clinical chemistry. Urine and blood samples were then centrifuged, separated into aliquots, and frozen at −80°C until analyzed. At the end of the study, rats were euthanized by exsanguination through cardiac puncture under isoflurane anesthesia (Isoflurane (5% for induction, then 1–2% for maintaining a steady state), and kidneys were collected for histology and bulk for RNA Sequencing (RNA-Seq) analysis.

Ethylenediamine tetraacetic acid (EDTA) blood samples were processed to plasma (approximately 10,000 RPM for 3–5 minutes). EDTA plasma samples were transferred using a pipette into a 2 mL 96-well plate, and frozen immediately over dry ice or in a freezer set to maintain -80°C. For clinical biochemistry assay, on the day of analysis the samples were treated with LipoClear (Thermo Fisher Scientific, Waltham, MA) after initial centrifugation (20 μL of LipoClear was added for every 100 μL of plasma). The LipoClear-treated aliquot was centrifuged in a micro-centrifuge for 2 minutes at 9800–10,500 rpm and then transferred to a fresh tube for analysis.

### Tissue collection and preservation

Representative samples of left kidney were collected and preserved in 10% neutral buffered formalin for 24–48 hours and then transferred to 70% alcohol. A portion of the right kidney was snap-frozen with liquid nitrogen prior to be placed into a cryovial. The snap-frozen kidney tissue was stored in a freezer set to maintain -80°C for further RNA-Seq analysis.

### Biomarker measurement and plasma compound level analysis

The following were measured by Roche Modular Chemistry System (Roche Diagnostics, Indianapolis, IN): Blood urea nitrogen (BUN), total protein, albumin, cholesterol, low-density lipoprotein (LDL), high-density lipoprotein (HDL), triglycerides (TG), glucose in plasma and urinary urea nitrogen, creatinine, total protein, albumin, and glucose in urine. Creatinine clearance (CrCL) was calculated using the following formula: CrCL = urinary creatinine excretion rate/plasma creatinine concentration. The following were also measured: Urinary albumin concentration and levels of kidney injury biomarkers, including kidney injury molecule-1 (KIM-1), Cystatin C, and neutrophil gelatinase-associated lipocalin (NGAL), an acute kidney injury biomarker. Plasma exposure of Enalaprilat and Compound 1 were determined by liquid chromatography-tandem mass spectrometry (LC-MS/MS) using a LC-MS/MS mass spectrometer 4500 Triple Quad (Sciex, Redwood City, CA). cGMP was measured using commercially available kit (cGMP ELISA kit item, #581021, Caymanchem Ann Arbor, MI).

### Tissue total RNA isolation and RNAseq sequencing

Kidney tissues were extracted from experimental animals and immediately snap-frozen in liquid nitrogen and stored at −80°C for analysis. Tissues were homogenized into RNA STAT-60 (Tel-Test Inc., Friendswood, TX) using a polytron homogenizer, followed by total RNA isolation using the MagMAX mirVana Total RNA isolation kit (Thermo Fisher Scientific Inc., Foster City, CA) according to the manufacturer’s instructions. Total RNA quality was quantified and qualified on the Agilent Fragment Analyzer System using the RNA standard sensitivity kit per manufacturer’s instructions (Agilent, Santa Clara, CA). Sequencing libraries were prepared using Illumina’s TruSeq Stranded mRNA kit per manufacturer’s instructions (Illumina, San Diego, CA) and sequenced on the Illumina Novaseq with 100 paired end read length and 8G output read depth.

### Kidney histology assessment

Kidney tissue was collected immediately at the end of the study and fixed by immersion in 10% phosphate-buffered formalin for 48 hours. Formalin-fixed tissues were washed in a phosphate buffer, dehydrated through a graded series of ethanol and xylene, and then paraffin-embedded. Tissue sections were stained with hematoxylin and eosin (H&E), periodic acid–Schiff (PAS), and Masson’s trichrome, and evaluated under a light microscope. The extent of histopathologic changes in renal tubules, interstitial, vasculature, and glomeruli were graded on a 0 to 5 scale corresponding to normal, minimal, mild, moderate, marked, and severe. Collagen deposition in the kidney was graded on a 0 to 5 scale corresponding to minimal, mild, moderate, marked, and severe, based on the size and intensity of the blue-stained area. The findings are separated for tubules, interstitial, vasculature, glomeruli, and fibrous tissue. Severity score 5 (severe) did not occur in this study. In addition, a combined severity score for each animal is provided as “nephropathy,” an “approximate estimate” combined score of the changes, not a statistical average.

### Pharmacokinetic measurements

Chromatographic separation was performed on an Acquity I-Class UPLC (Waters, Milford, MA). Samples without polyethylene glycol in the dosing solution were analyzed with a Waters HSS T3 1.8 μm, 2.1 x 50 mm column maintained at 40°C with a flow rate of 0.75 mL/min. Mobile phase A consisted of water with 0.1% formic acid, and mobile phase B consisted of acetonitrile with 0.1% formic acid. An initial condition of 95% A was held for 0.25 minutes, followed by a linear gradient to 95% B for 1.5 minutes, held at 95% B for 0.4 minutes, and then equilibrated back to 95% A.

Samples with polyethylene glycol in the dosing solution were analyzed with a Phenomenex Kinetex Biphenyl 2.6 μm, 2.1 x 50 mm column (Phenomenex, Torrance, CA) maintained at 40°C with a flow rate of 0.75 mL/min. This alternate separation was required due to coelution and ionization suppression of Enalaprilat by PEG with the HSS T3 method. Mobile phase A consisted of water with 0.1% formic acid, and mobile phase B consisted of methanol with 0.1% formic acid. An initial condition of 60% A was held for 0.25 minutes, followed by a linear gradient to 60% B for 1.5 minutes, increased to 95% B and held for 0.4 minutes, and then equilibrated back to 60% A.

Mass spectrometer quantitation was performed on a 4500 Triple Quad (Sciex) with electrospray ionization under positive mode with Analyst 1.6.3 operating software. The following ion transitions were used: Enalaprilat (349.0/206.0, DP 71, CE 25) and Compound 1 (537.2/509.3, DP 100, CE 55).

For the Sample preparation, calibrators for Enalaprilat and Compound 1 were dispensed into individual wells by an HP D300 Digital Dispenser (HP, Palo Alto, CA) with final concentrations from 1 nM to 10 μM after addition of 50 μL blank ZSF1 OB rat plasma. 40 μL calibrators and study samples were protein- precipitated with a 160 μL internal standard cocktail (200 nM labetalol, 200 nM imipramine, and 200 nM diclofenac in acetonitrile with 0.1% formic acid) and passed through a Waters Ostro plate for phospholipid removal. The filtrate was mixed with an equal volume of water with 0.1% formic acid and analyzed by liquid chromatography with tandem mass spectrometry (LC-MS/MS).

### Statistical analysis

All data are presented as mean ± SEM. Data collected repetitively over time were analyzed using a two-way analysis of variance with repeated measures and a Tukey post hoc analysis using GraphPad Prism Version 8.0 (GraphPad Software, La Jolla, CA).

Data such as biomarkers and histologic endpoints were analyzed using a one-way analysis of variance with Dunnett’s post hoc analysis. A *P* value of <0.05 is statistically significant.

### Gene expression analysis

#### Kidney transcriptional analysis

Forty-four kidney samples were used for transcriptional analysis, with 11 vehicles, 9 treated with Enalapril, 12 treated with Compound 1, and 12 treated with a combination of Enalapril and Compound 1. Prior to analyzing the count matrices, a pre-processing filtering was applied to remove low expression genes by requiring each gene’s fragments per kilobase of transcript per million mapped reads (FPKM) value to be >1 in at least 9 samples. Raw count files for each treatment vs vehicle group were used as input of DESeq2 in R to identify differentially expressed genes (DEGs). We considered DEGs significant if their adjusted *P* values were <0.05 and their fold change was >1.50 (up-regulation) or less than <0.66 (down-regulation).

#### Pathway enrichment analysis

To get a comprehensive view of significantly regulated pathways, we first identified enriched Kyoto Encyclopedia of Genes and Genomes (KEGG) rat pathways using a clusterProfiler R package based on up- and down-regulated DEGs selected from each of the treatment vs vehicle group. Only pathways that with members >5 and <500 were considered. We next used QIAGEN’s Ingenuity Pathway Analysis (IPA®, QIAGEN Redwood City, CA) to identify cellular processes associated with the identified DEGs. In both cases, significant pathways (*P* adj <0.05) were further investigated.

## Results

### Effect of compound 1 to activate sGC in functional CASA assay

**Fig 1:**
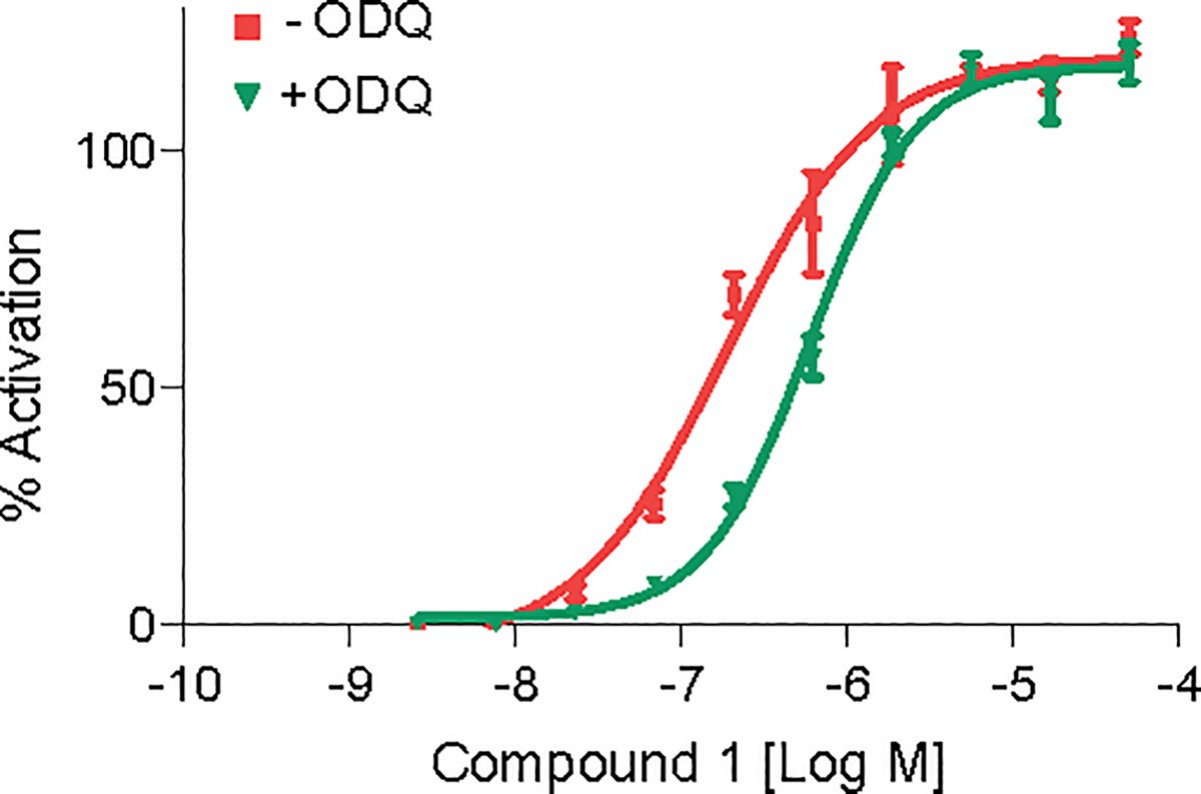
In vitro activity of compound 1 in CHO-K1/sGC stable cell line. The in vitro activity of compound 1 was assessed in cell-based sGC Functional Assay (CASA Assay) using CHO-K1/sGC stable cell line. Red square denotes compound 1 sGC activation in absence of ODQ and the green triangle shows activation in presence of ODQ.

Compound 1 increased sGC activity in recombinant CHO-K1 stable cell line expressing sGC, when the heme group is in ferrous state or oxidized to ferric state by treatment with ODQ as shown in **Fig 1**. Compound activates sGC with potency of (EC_50_ = 135.1±1.18 nM) without ODQ and (EC_50_ = 543.1±1.08 nM) with ODQ, respectively.

### Acute effects of Compound 1 on blood pressure and RBF in anesthetized CD rats

**Fig 2:**
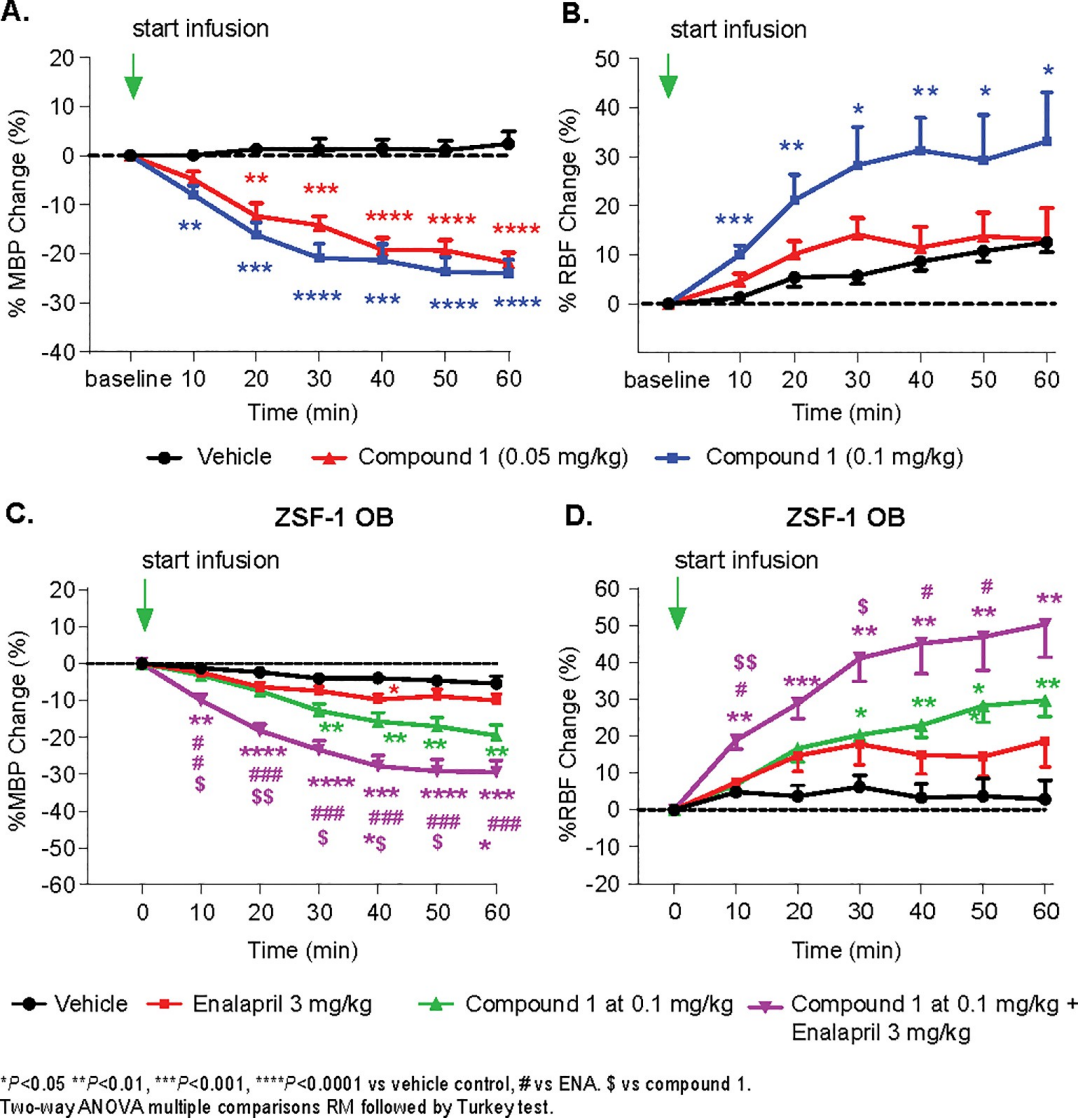
Acute effect of Compound 1 on MBP and RBF in anesthetized CD rats. Green arrow indicates start of IV infusion of Vehicle or Compound 1 for 30 minutes. (A) Black circle represents data from vehicle treated group; red triangle represents data from low dose of compound 1 at 0.05 mg/kg, the blue rectangle represents high dose of compound 1 at 0.1 mg/kg. Each data point represents the averaged data of MBP in a 10-min interval and shows dose-dependent effect of Compound 1 on the percentage change of MBP from baseline. In comparison with data from the Vehicle-treated group, Compound 1 significantly decreased MBP in both 0.05 and 0.1 mg/kg doses.(B) shows dose-dependent effect of Compound 1 on the percentage change of RBF. Only the high dose of Compound 1 significantly increased RBF vs Vehicle control. *P <0.05 **P <0.01, ***P <0.001, ****P <0.0001 vs Vehicle control. Two-way repeated measures ANOVA with multiple comparisons followed by Tukey test. C and D. Acute effect of Compound 1 and/or Enalaprilat on MBP and RBF in anesthetized ZSF1 OB rats.

Compound 1 induced a dose-dependent effect on BP reduction: 21.8 ± 2.1% and 24.0 ± 2.8% at the 60-minute time point for 0.05 mg/kg and 0.1 mg/kg, respectively **(Fig 2A)**. However, RBF was significantly increased only in the high-dose treated group (33.8 ± 7.3%) and not in the low-dose one (13.2 ± 6.4% vs 12.6 ±2.0% in vehicle control group) despite a similar BP drop (21.8 ± 2.1% vs 24.0 ± 2.8%) at 60 minutes **(Fig 2B)**.

### Acute effects of Compound 1 on BP and RBF in anesthetized ZSF1 OB rats

When both Compound 1 and positive control Enalaprilat were administered to ZSF1 OB rats, Enalaprilat only reduced BP moderately (10.0 ± 1.8% vs. 5.4 ± 2.0 in vehicle control), while Compound 1 significantly reduced BP for 19.5 ± 2.9% in ZSF1 OB. The additive effect on BP reduction (29.4 ± 3.1%) was observed in the combination treatment group **(Fig 2C)**. Enalaprilat only moderately increased renal blood flow (18.6 ± 7.0% vs. 2.9 ± 5.1% in vehicle control, which was not significant). Compound 1 significantly increased RBP 29.6 ± 4.3% and the combination treatment had a synergistic effect on RBF of 50.3 ± 8.9% at 60 minutes **(Fig 2D)**. At 60 minutes, the plasma concentration of Compound 1 is 356.0 ± 43.2 nM and the concentration of Enalaprilat is 34.5 ± 4.8 μM.

Green arrow indicates start of IV infusion of Vehicle or Compound 1 or Enalaprilat or combination of Compound 1 and Enalaprilat for 30 minutes. Black circle represents data from vehicle treated group; red rectangle represents data from enalaprilat treated group; green triangle represents of compound 1 at 0.1 mg/k while purple triangle represents that of combination treatment of both enalaprilat and compound 1 at 0.1 mg/kg. (C) Each data point represents the averaged data of MBP in a 10-min interval and shows the effect of Compound 1, Enalaprilat or combination of both on the percentage change of MBP from baseline. In comparison with the data from the Vehicle-treated group, Enalaprilat moderately reduced BP, Compound 1 significantly decreased MBP, and a combination of Compound 1 and Enalaprilat showed additional BP reduction. (D) shows the effect of treatments on the percentage change of RBF vs Vehicle control. Enalaprilat did not change RBF and Compound 1 significantly increased RBF. The combination of Compound 1 and Enalaprilat showed additive or synergistic effects on RBF. *P <0.05 vs Vehicle control, # vs ENA. $ vs Compound 1. Two-way repeated measures ANOVA with multiple comparisons followed by Tukey test.

#### Chronic effect of sGC stimulators on systemic hemodynamics

**Fig 3:**
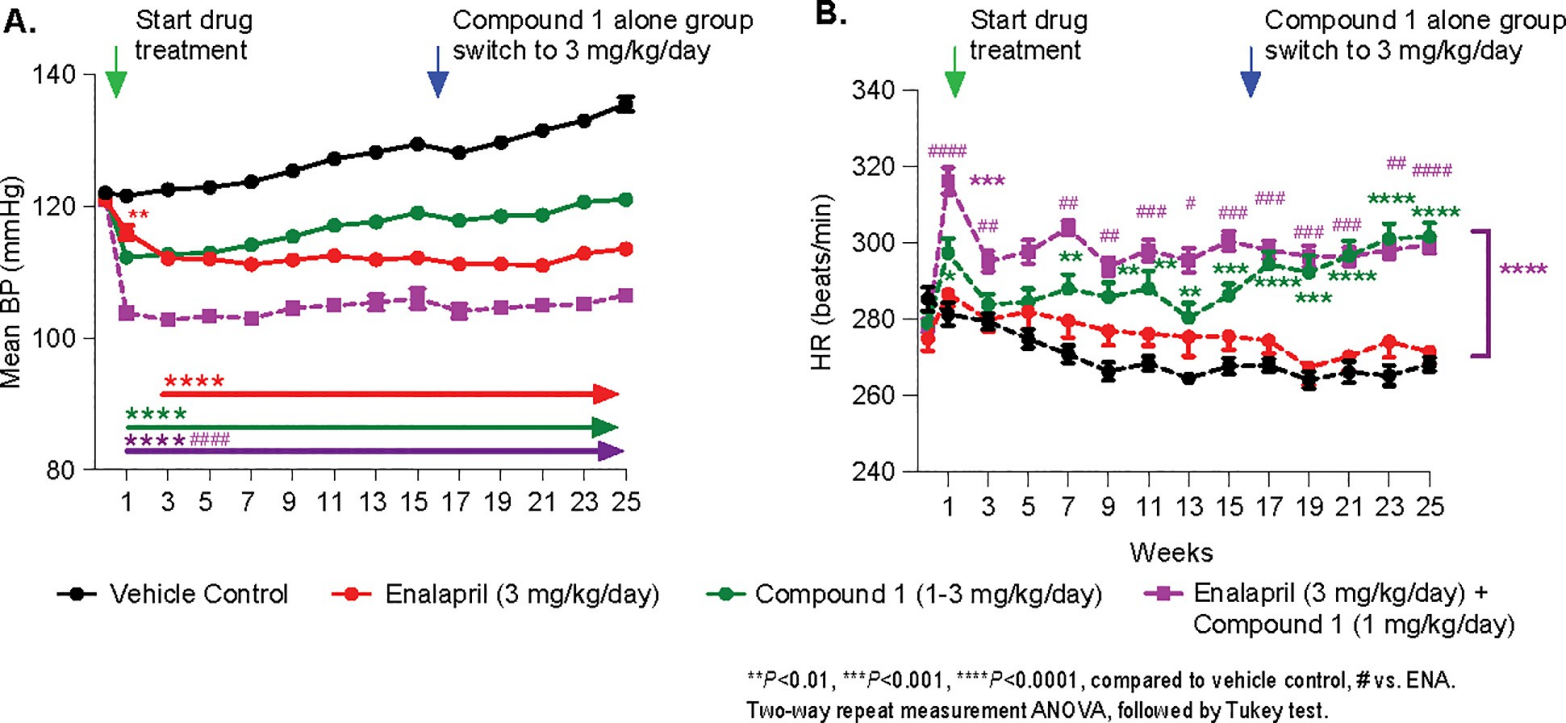
Effect of Compound 1 or Enalapril or combination of Compound 1 and Enalapril on MBP and HR. Green arrow indicates start of the treatments of Vehicle or Compound 1 or Enalapril or combination of Compound 1 and Enalapril for 6 months. Blue arrow indicates Compound 1 dose was switched from 1 mg/kg/day to 3 mg/kg/day. (A) Compound 1 significantly reduced MBP but not as much as Enalapril treatment, while a Compound 1 and Enalapril combination reduced MBP to normotensive level. (B) HR was significantly increased in both Compound 1-treated group and combination-treated groups. Two-way repeated measures ANOVA with multiple comparisons followed by Tukey test.

Because the effect of Compound 1 on BP was normalizing, the dose of Compound 1 was increased from 1 mg/kg/day to 3 mg/kg/day in order to achieve a similar degree of BP reduction seen in the Enalapril-treated group. Compound 1 significantly reduced MBP (-10.7± 0.7% vs vehicle control group). Enalapril reduced MBP more (-16.2 ± 0.5% vs vehicle control group), while a combination of Compound 1 and Enalapril reduced MBP (-21.4 ± 0.6% vs vehicle control group) to a near normotensive level at week 25 **(Fig 3A)**. HR was significantly increased by Compound 1 (12.5 ± 1.4% vs vehicle control) and increased to 11.7 ± 0.9% in the combination-treated groups **(Fig 3B)**. The positive control Enalapril at 3 mg/kg/day had almost no effect on HR (1.2 ± 1.1%). Plasma exposure of Compound 1 is 72.3 ± 5.2 nM and the Enalaprilat concentration of the Enalapril metabolite is 215.8 ± 11.4 nM.

### Chronic effect of sGC stimulators on renal function in ZSF1 OB rats

**Fig 4:**
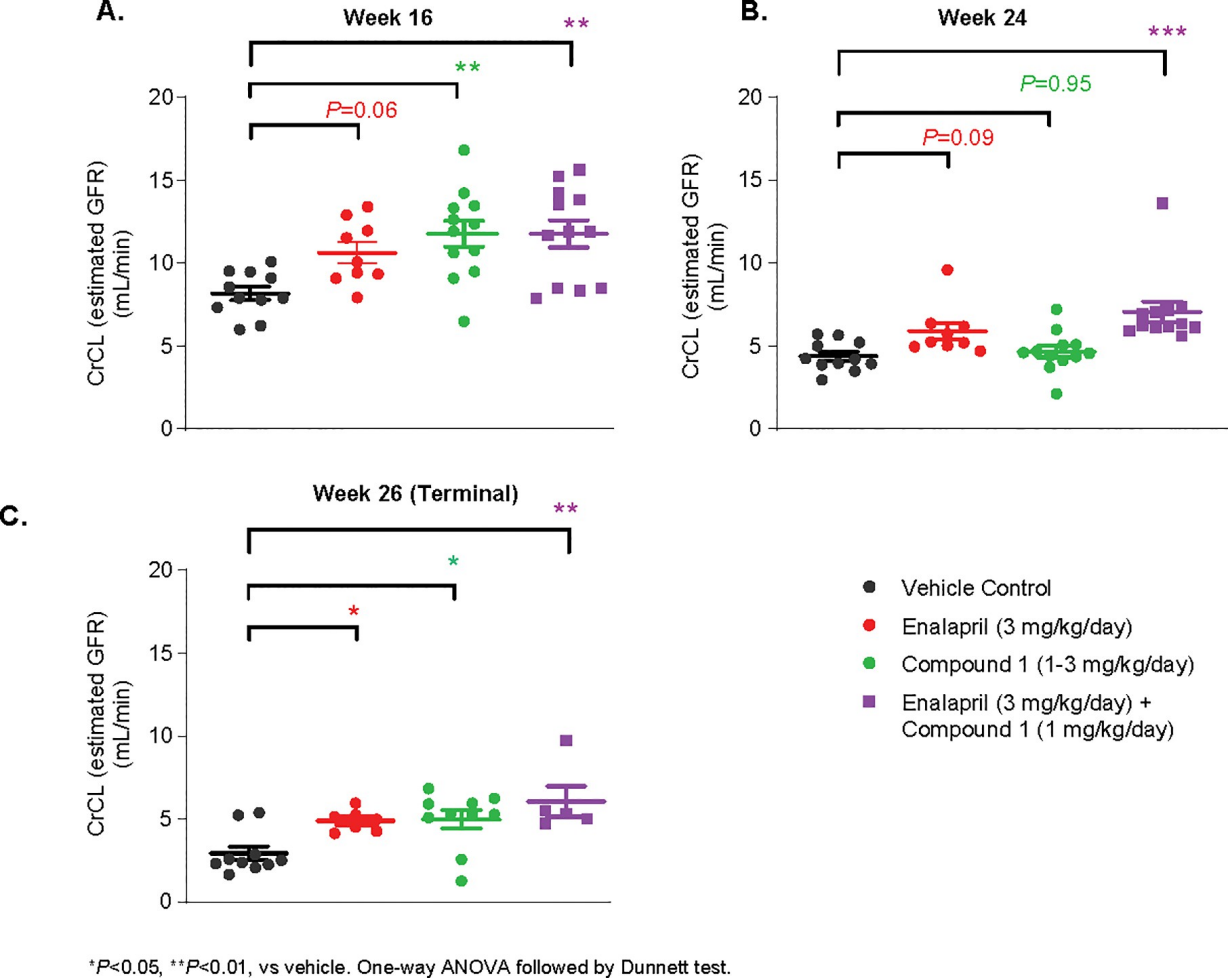
Chronic effects of Compound 1 on GFR in ZSF1 OB rats. GFR by creatinine clearance (CrCL) was improved by Enalapril, Compound 1 and combination at 16 weeks (A) and at (C) 26 weeks vs that of Vehicle control. *P <0.05 vs Vehicle control. One-way ANOVA followed by Dunnett’s test.

GFR, via CrCL, was improved by Enalapril, Compound 1 and a combination by 29%, 44% and 44%, respectively, at week 16. However, at week 24, a further increment of RBF in Enalapril (34%) or Compound 1 (7%) was observed compared to vehicle control. Only in the combination group was GFR increased significantly (61%). At 26 weeks, all treatment improved GFR compared to that of vehicle control: Enalapril improved 67%, Compound 1 improved 72% and the combination treatment improved by 110% **(Fig 4A–4C)**.

**Fig 5:**
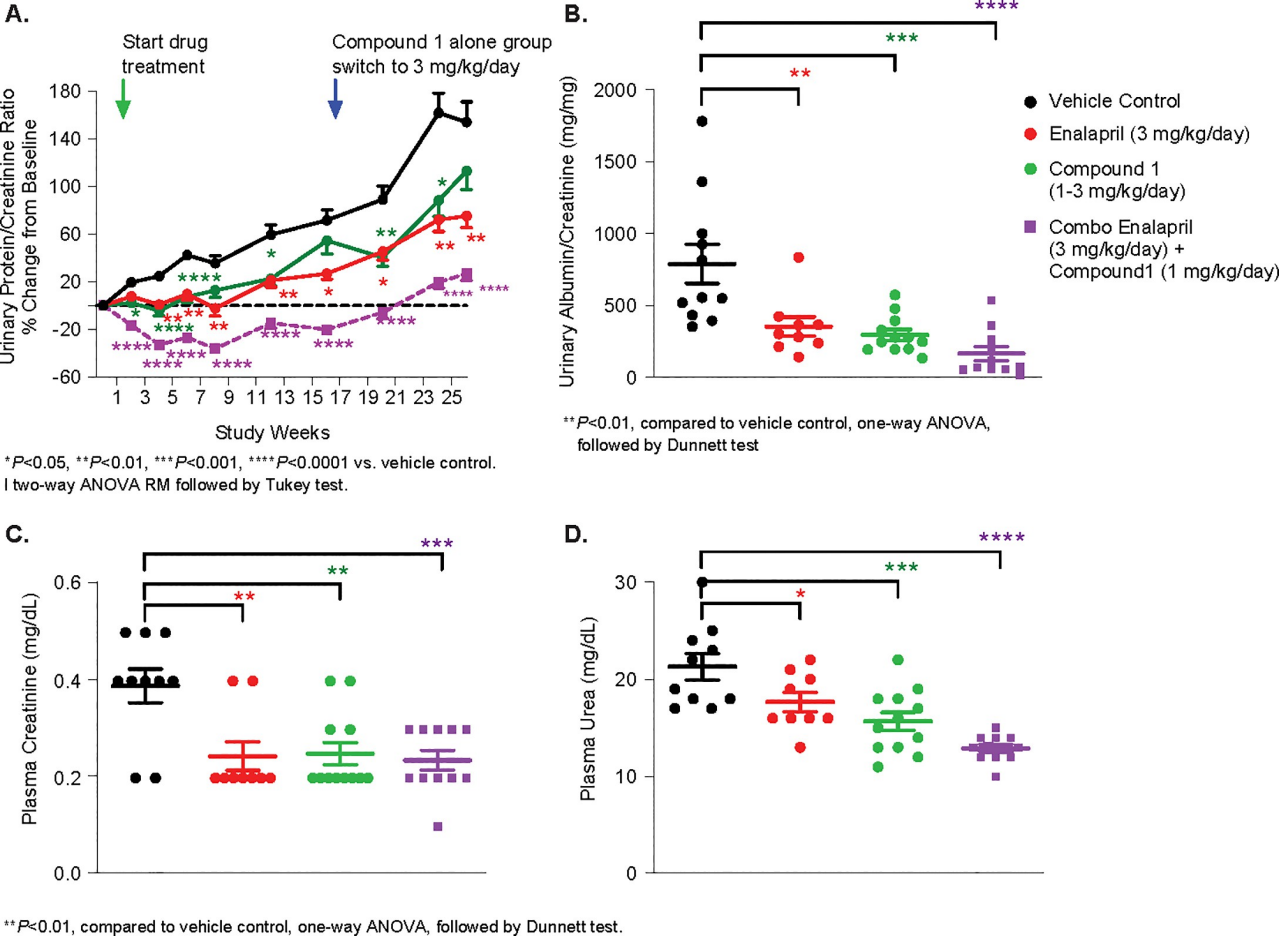
Effect of Compound 1 on urinary protein to creatinine ratio (UPCR). Green arrow indicates start of the treatments of Vehicle or Compound 1 or Enalapril or combination of Compound 1 and Enalapril for 6 months. Blue arrow indicates Compound 1 dose was switched from 1 mg/kg/day to 3 mg/kg/day. (A) Enalapril, Compound 1 and combination treatment significantly reduced protein to creatinine ratio compared to that of Vehicle control and (B) Albumin to creatinine ratio (UACR) at week 26. Enalapril, Compound 1 and combination of both significantly reduced albuminuria compared to that of Vehicle control. *P <0.05, **P <0.01, ***P <0.001, ****P <0.0001 vs Vehicle control. Two-way repeated measures ANOVA followed by Tukey test. Effect of Compound 1 on plasma creatinine and urea. Plasma levels of creatinine (5C) and urea (5D) were significantly reduced in all 3 treatment groups. **P <0.01, compared to Vehicle control, one-way ANOVA, followed by Dunnett’s test.

Obese ZSF1 rats developed proteinuria progressively over time. Enalapril, Compound 1 and the combination treatment significantly reduced protein to creatinine ratio by 63%, 27% and 83% compared to that of vehicle control **(Fig 5A)**. At week 26, Enalapril, Compound 1 and the combination significantly reduced albuminuria by 55%, 62% and 79%, respectively, compared to that of vehicle control **(Fig 5B)**.

Consistent with eGFR and urinary protein/albumin excretion, plasma levels of creatinine and urea were significantly reduced in all 3 treatment groups **(Fig 5C and 5D)**.

#### Plasma and urinary cGMP

All three-treatment group had similar plasma cGMP seen in that in vehicle treated group. Both compound 1 and combination of compound 1 plus enalapril significantly increase urinary cGMP (nmol/24 hour).

#### Urinary biomarkers

**Fig 6:**
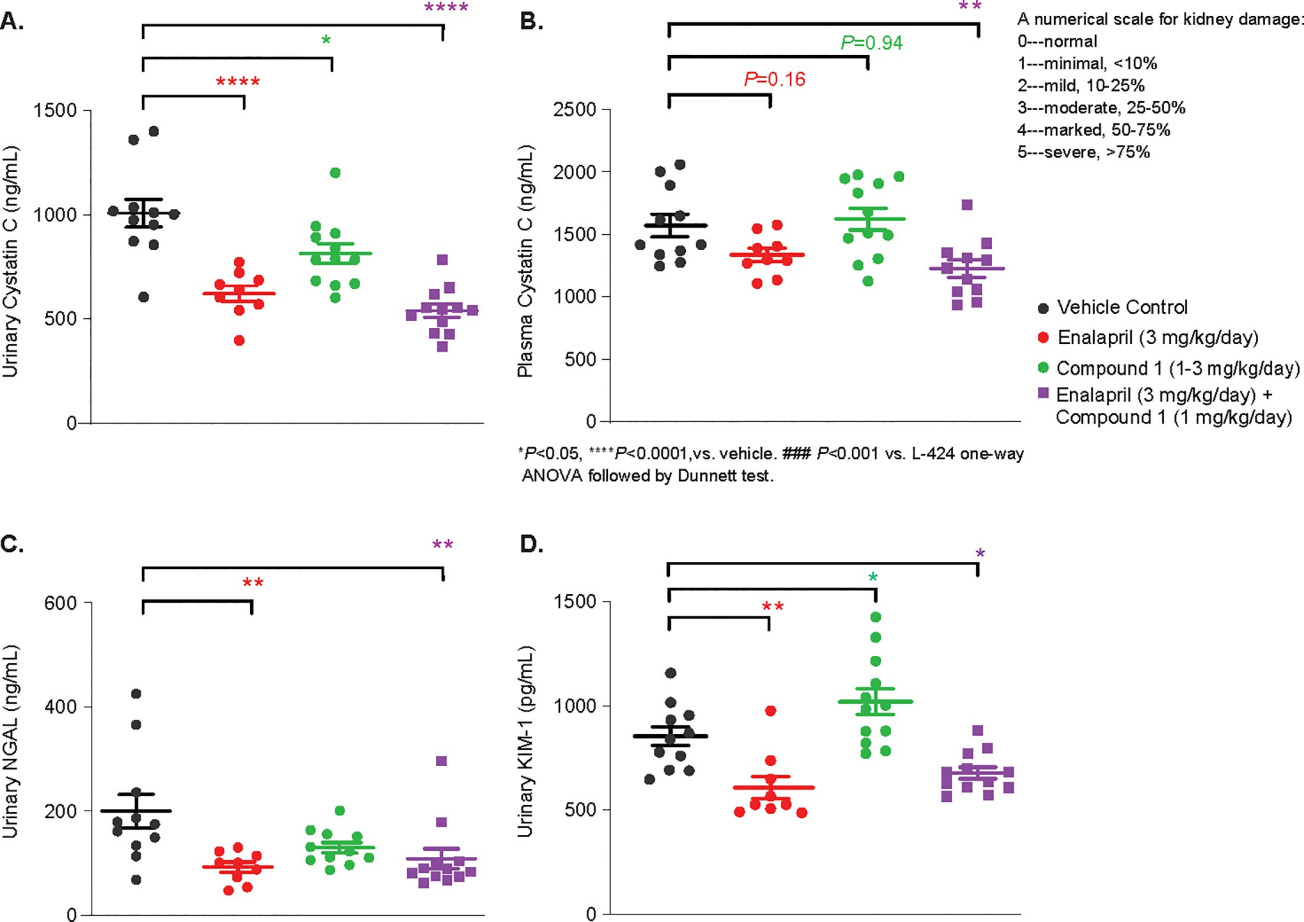
Effect of Compound 1 on plasma Cystatin C and urinary biomarkers. All 3 treated groups had reduced urinary Cystatin C concentration. (A) Only combination treatment significantly reduced plasma Cystatin C level. (B) Both Enalapril and combination treatments also reduced urinary NGAL (C) and (D) KIM-1. *P<0.05, compared to Vehicle control. One-way ANOVA, followed by Dunnett’s test.

All three treated groups had reduced urinary Cystatin C but only the combination treatment significantly reduced plasma Cystatin C levels **(Fig 6A and 6B)**. Both Enalapril and the combination treatment also reduced urinary NGAL and KIM-1 **(Fig 6C and 6D)**.

#### Renal histopathology

**Fig 7:**
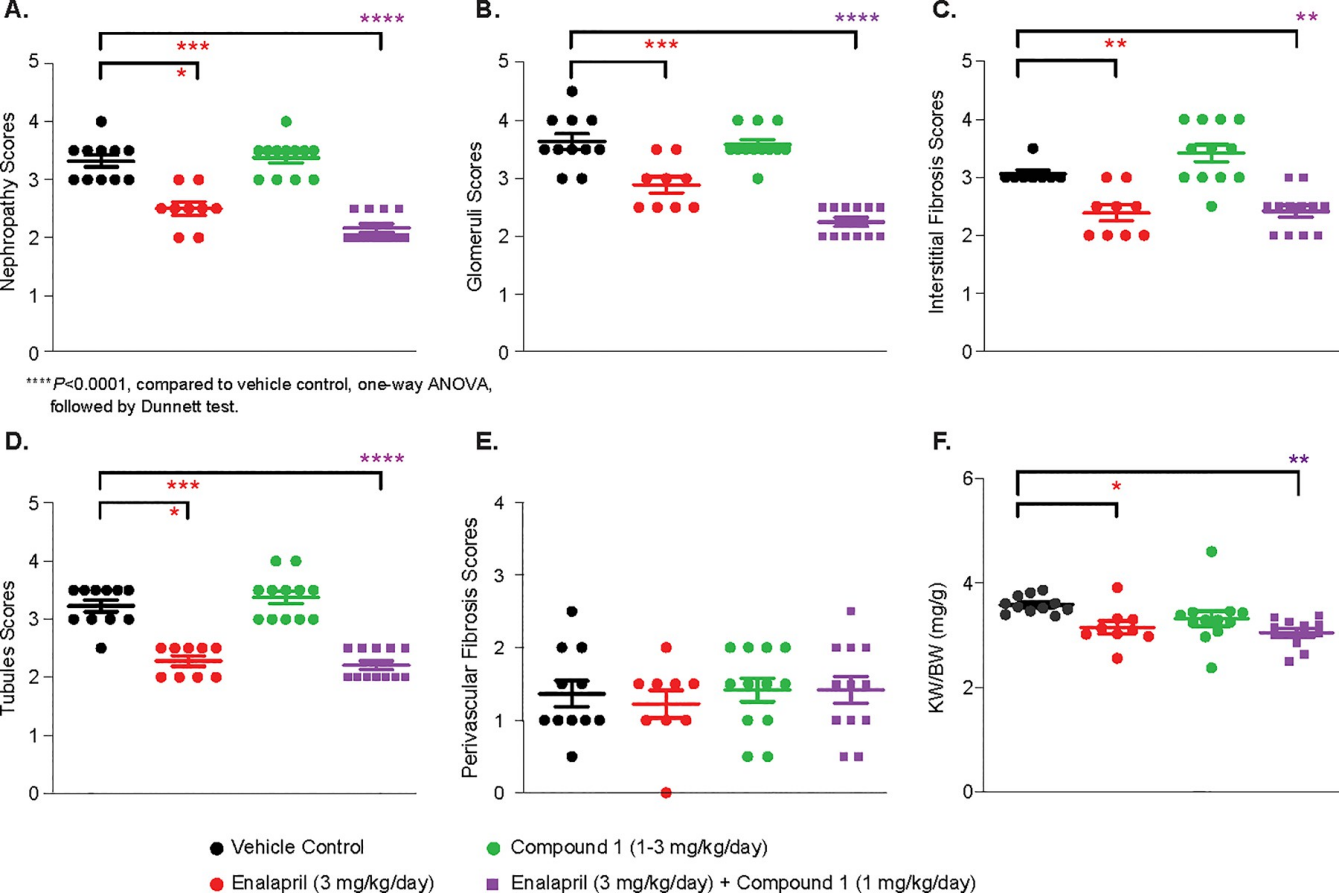
Renal histology after 6 months of Compound 1 administration. (A) Nephropathy score is a combined score from each component (tubules, interstitial, and glomeruli). Enalapril or combination significantly reduced nephropathy score as well as tubular injury (D) vascular interstitial (E) and glomerular injury scores (B), while Compound 1 did not have an effect. (F) Kidney weight to body weight ratio was significantly reduced by Enalapril or combination treatment.

A nephropathy score is a combined score from each component (tubules, interstitial, and glomeruli). Enalapril and the combination treatment significantly reduced nephropathy score as well as tubular injury, vascular interstitial and glomerular injury scores, while Compound 1 did not have any observable effect **(Fig 7A–7D)**. Consistent with renal histology, the kidney to body weight ratio was reduced with Enalapril treatment or the combination treatment **(Fig 7F)**, indicating a remodeling effect in the kidney.

### Gene expression profiling

**Fig 8:**
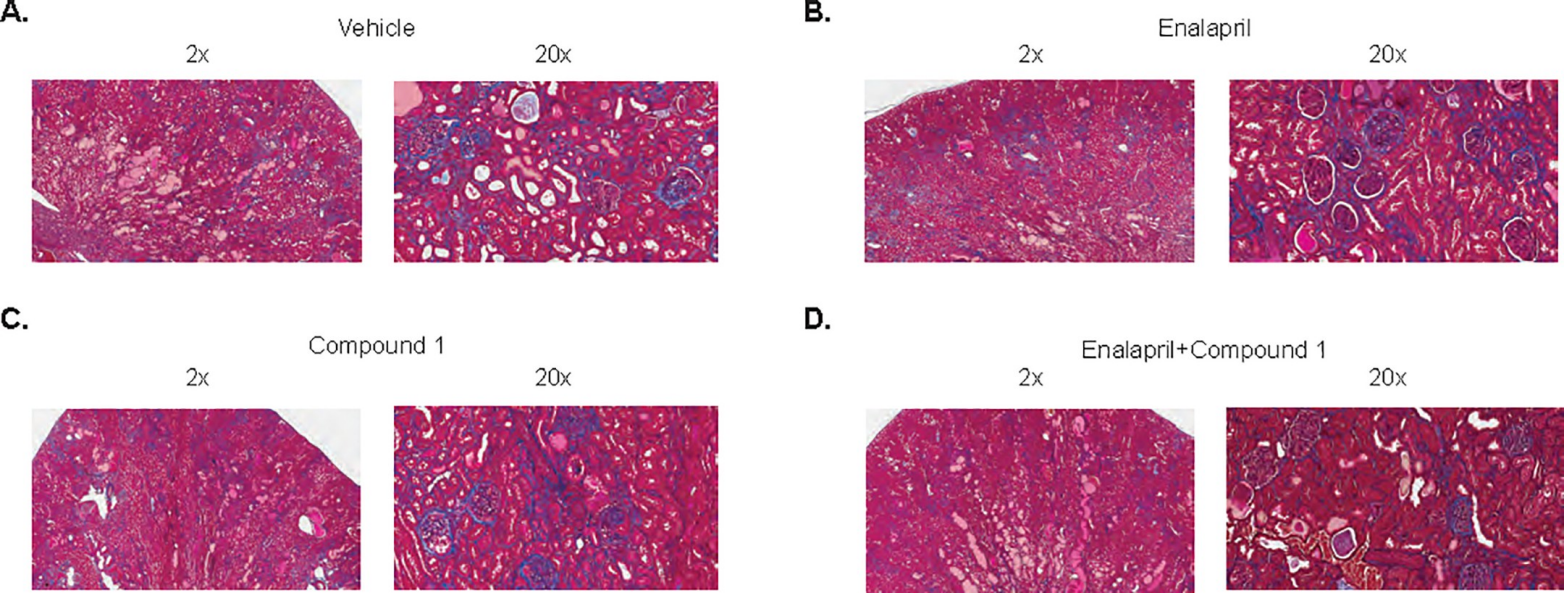
Representative histology imagines (Masson’s Trichrome). Trichrome imagines are proved at 2x and 20x magnification. (A) Vehicle treated; (B) Enalapril treated; (C) Compound 1 treated and (D) combination of enalapril and compound 1 treated groups.

There are 379, 38 and 652 DEGs in the Enalapril vs Vehicle, Compound 1 vs Vehicle, and Enalapril and Compound 1 vs Vehicle groups, respectively, with the most significant 20 DEGs in the 3 groups **(Fig 8A–8C)**. In the Enalapril vs Vehicle and Compound 1 vs Vehicle groups, more than half of the top 20 genes were down-regulated, while in the combination treatment group, up- and down-regulated genes were evenly distributed from the top 20 DEGs. The FPKM values of up-regulated genes among the top 20 from Compound 1 vs Vehicle group are shown in **Fig 8D**. These values include the following genes: *FA2H* (fatty acid 2-hydroxylase, Sphingolipids pathway); *ESRRB* (human estrogen-related receptor beta); *SLC23A3* (electrochemical potential-driven transporters); *PGAM2* (glycolytic enzyme); *EGF* (the epidermal growth factor receptor EGFR signaling pathway); *CD163* (a high-affinity scavenger receptor for the hemoglobin-haptoglobin complex, a marker of cells from the monocyte/macrophage lineage; *MATN1* (cartilage matrix protein; and *REN* (renin, part of the renin-angiotensin system).

### Functional change

**Fig 9:**
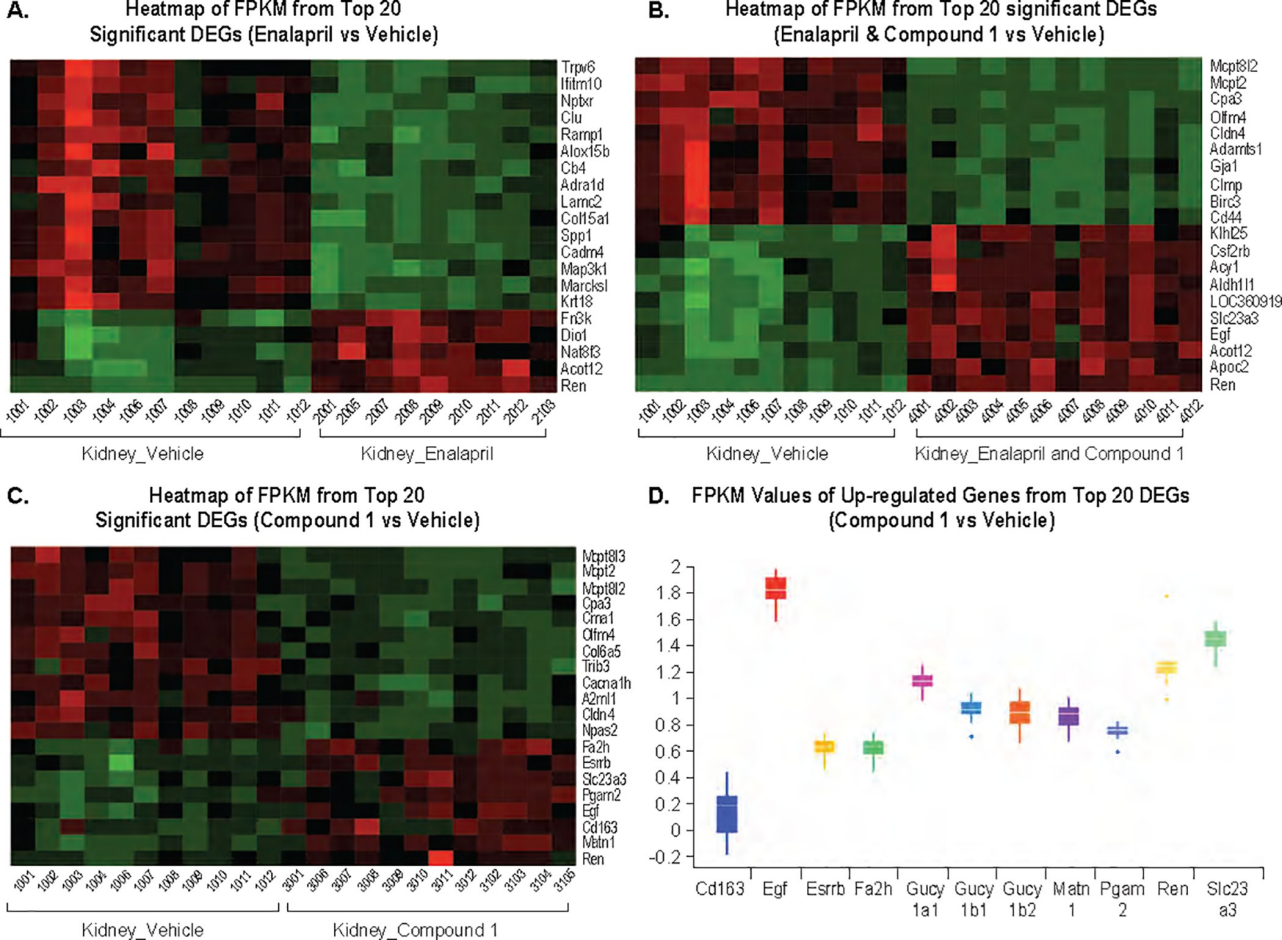
Significant differentially expressed genes. (A) Heatmap of most significant 20 genes from Enalapril vs Vehicle. (B) Heatmap of most significant 20 genes from Enalapril and Compound 1 vs Vehicle. (C) Heatmap of most significant 20 genes from Compound 1 vs Vehicle. (D) Upregulated genes from most significant 20 genes from Compound 1 vs Vehicle.

**Fig 10:**
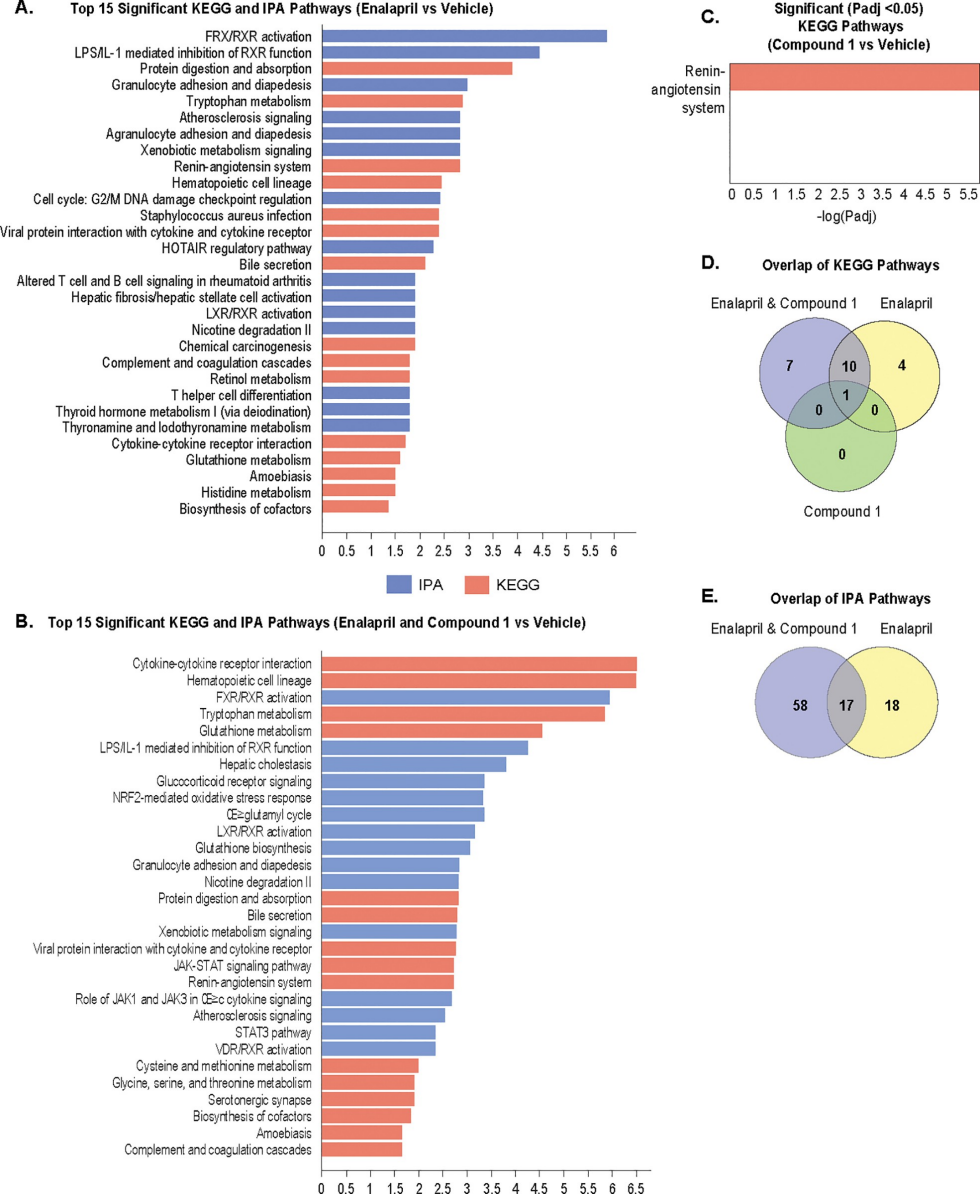
Significantly enriched pathways. (A) Top 15 significant pathways of both KEGG and IPA from Enalapril vs Vehicle. (B) Top 15 significant pathways of both KEGG and IPA from Enalapril and Compound 1 vs Vehicle. (C) Significant (*P* adj <0.05) KEGG pathways from Compound 1 vs Vehicle. (D) Venn diagram of significantly enriched KEGG pathways (*P* adj <0.05) from 3 treatments. (E) Venn diagram of significantly enriched IPA pathways (*P* adj <0.05) from Enalapril and Compound 1 vs Vehicle and Compound 1 vs Vehicle.

To determine the mechanisms regulated by different treatments, we studied the significantly enriched pathways from both KEGG and IPA, and the top 15 of both KEGG and IPA enriched pathways from each of the treatment groups are shown in **Fig 9A–9C**. Specifically for the combination therapy, the significantly enriched IPA pathways are FXR/RXR activation, LPS/IL-1–mediated inhibition of RXR function, hepatic cholestasis, NRF2-mediated oxidative stress and glucocorticoid receptor signaling, while for the significantly enriched KEGG pathway they include the renin-angiotensin system, cytokine-cytokine receptor interaction, tryptophan and glutathione metabolism and the Janus kinase/signal transducers and activators of transcription (JAK-STAT) pathway. Compound 1 monotherapy doesn’t yield a functional change due to the small set of DEGs associated with it. Overlapping pathways indicate that combination therapy and Enalapril monotherapy share many treatment-induced phenotypic changes, though combination therapy showed more differentially expressed genes as shown in **Fig 10**.

## Discussion

Given the critical role and the broad impact of the NO-sGC-cGMP pathway on vasculature and on multiple organ systems in health and disease, sGC stimulators have been investigated for cardiovascular cardiorenal and cardiopulmonary disease therapy [34, 35, 37–39]. Riociguat is a first-in-class sGC stimulator approved in 2013 for the treatment of pulmonary arterial hypertension (PAH) and chronic thromboembolic pulmonary hypertension (CTEPH). In addition to its effects in PAH, riociguat was able to reduce blood pressure which could offer benefit in patients resistant to angiotensin ll receptor treatment [32]. Riociguat single treatment could not reduce albuminuria significantly, however combination with telmisartan reduced urinary albumin excretion in STZ treated eNOS KO mice. Sharkovska et al., has reported riociguat can improve survival and normalize blood pressure in L-NAME (high-renin model). In this study compound 1 also an sGC stimulator can also normalize hypertension by lowering the mean blood pressure, albeit maintaining heart rate and increasing renal blood flow in two in vivo models. Riociguat improved renal endpoints such as lowering plasma creatinine, reduced glomerulosclerosis, and less renal interstitial fibrosis in low and high renin-models. Similar renal benefits were observed with Compound 1 treatment alone and significant effects on top of Enalapril in reducing target organ damage.

Recently, a successful clinical trial reported that when compared with placebo, Vericiguat, a novel oral sGC stimulator, significantly reduced the incidence of cardiovascular death and hospitalization for heart failure (HF) in patients with worsening HF with reduced ejection fraction (HFrEF) [35]. The growing evidence from both preclinical and clinical standpoints suggests a critical role for NO-sGC-cGMP signaling in kidney pathophysiology and impaired cardiorenal crosstalk. Down-regulation of NO-sGC-cGMP is implicated in the pathogenesis of CKD [1]. cGMP directly influences renal blood flow, renin secretion, glomerular function, and tubular exchange processes. Therefore, treatment strategies aiming to stimulate or activate the NO-sGC-cGMP pathway might have beneficial effects for the treatment of progressive kidney diseases. Here we investigated the effects of an sGC stimulator invitro and in vivo to investigate the renal function and histopathology in a preclinical DN model.

The ZSF1 obese rat model exhibits common molecular pathway dysregulation in DN, such as reduced renal NO production and increased renal reactive oxygen species [1]. Indeed, the ZSF1 OB rat is one of very few rodent models of DN that meets the criteria of rodent models of DN by The Animal Models of Diabetic Complications Consortium (AMDCC) [40]. At 8 weeks, obese ZSF1 rats developed metabolic syndrome and hypertension and early signs of renal disease, including proteinuria, glomerular collagen IV deposition, tubulointerstitial inflammation, and renal hypertrophy. By 32 weeks, animals developed renal histopathology consistent with DN, including mesangial expansion, glomerulosclerosis, tubulointerstitial inflammation and fibrosis, tubular dilation and atrophy, and arteriolar thickening [27]. In this study, we investigated Compound 1 alone and in combination with standard of care drug Enalapril to understand the renal effects on function, remodeling and biomarkers. Our data strongly suggest that Compound 1 monotherapy improves renal function, but the combination of Compound 1 and Enalapril has greater benefit in many aspects of renal function and renal histopathological scores. Therefore, Compound 1 and Enalapril combination therapy could be protective against DN that is associated with normalized blood pressure, attenuating the deterioration of GFR and albuminuria, and reducing interstitial fibrosis and glomerulosclerosis.

It is well established that sGC modulators stimulate the signal cascade of NO-sGC-cGMP and induce vasodilatory effects by increasing cGMP [41]. Stimulators and activators of sGC target the enzyme in two different redox states: the NO-sensitive reduced (ferrous) enzyme and NO-insensitive oxidized (ferric) enzyme, and finally heme-free enzyme, respectively. Stimulators of sGC stimulate the reduced form of sGC and synergize with available NO. Conversely, sGC activators increase the activity of the enzyme only when the heme iron is oxidized which subsequently lead to the heme-free enzyme. They bind to the unoccupied heme-binding complex and produce only an additive effect with NO. Invitro Compound 1 exhibited dual mode of action including directly stimulating the sGC independent of NO also sensitizing sGC, increasing cGMP production when the heme group is in ferrous state or oxidized to ferric state. We believe the Compound 1 unique profile in its ability to stimulate sGC in the absence or presence of NO, can have beneficial effects in attenuating CKD progression. Our data with compound 1 is supported by literature showing similar mechanism of action with sGC stimulators [38]

**Fig 11:**
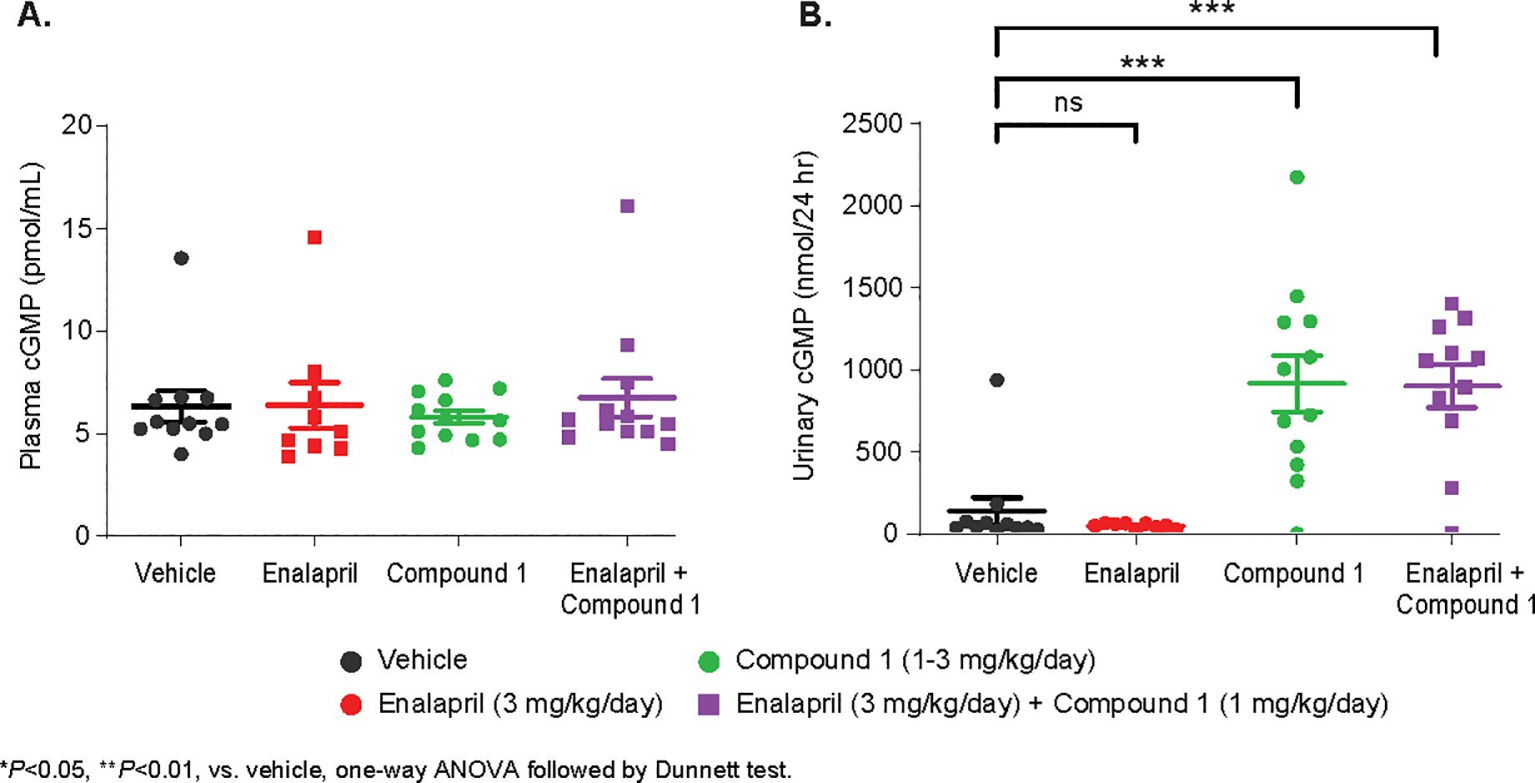
Effect of Compound 1 on plasma and urinary cGMP. Plasma levels of cGMP (A) are similar among treatment group. Urinary cGMP (B) is significantly increased in compound 1 treated alone or combination of compound 1 and enalapril vs. that in vehicle control treated. **P <0.01, compared to Vehicle control, one-way ANOVA, followed by Dunnett’s test.

Chronic treatment of Compound 1 significantly increased cGMP level in urine while the plasma cGMP level did not change comparing to vehicle or Enalapril treatment alone (**Fig 11**). We also observed enhanced expression of sGC subunit genes upon treatment with compound 1 from RNASeq analysis (**Fig 9D**). Urinary cGMP is of renal cellular origin after glomerular filtration and produced locally as a result of the action of sGC modulation on the local collecting ducts. Because the plasma level of cGMP is similar among the treatment group, the higher level of urinary cGMP is most likely contributed by increase the secretion of cGMP from the collecting duct, correlating with the natriuresis induced by ANP. Moreover, urinary cGMP is proposed as a biologic marker for renal activities. The lack of cGMP effect in plasma is not surprising due to major contribution from endogenous natriuretic peptides and only minor from other pathways such as soluble guanylate cyclase. Direct effect of the Compound 1 in increasing urinary cGMP and not plasma treatment indicates the target engagement of renal origin. This was further confirmed by QPCR and RNAseq analysis demonstrating the enhanced expression of sGC subunit genes upon treatment with compound 1 (S1 Fig). Compound 1 modulates sGC and cGMP pathway, and consequently regulate the hemodynamic response. As a result, Compound 1 reduced blood pressure in normal CD rats as well as ZSF1 obese rats. The blood pressure rapidly dropped, and then gradually bounced back with chronic treatment of Compound 1. However, the combination of Compound 1 and Enalapril showed a consistent and robust additive effect normalized to MBP similar to that observed in ZSF1 lean rats. The initial blood pressure-lowering effect may be due to cGMP counteracting renin-angiotensin II–induced vasoconstriction [42]. Activation of sGC can induce many pathways and one of effects is through AT1 receptor. Our RNA-Seq data confirmed that the renin-angiotensin system pathway was significantly enriched after Compound 1. This is in concordance with literature reports that sGC stimulators increases renin expression & secretion by cGMP crosstalk via PDE3-mediated cAMP [43, 44]. In addition, we observed expression of protein-kinase G ll by compound 1 indicating strong involvement on renin release as reported for other sGC stimulators [45]. Perhaps this is the reason why chronic treatment of compound 1 alone could not sustain the blood pressure lowering effect but the combination of Compound 1 and Enalapril, an angiotensin converting enzyme (ACE) inhibitor, counterbalances the renin-angiotensin II -dependent activation of the AT1 receptor by Compound 1. An increase of cGMP levels by Compound 1 may offset angiotensin-induced Na^+^ reabsorption in the proximal tubule, which suggests a blood pressure-lowering mechanism independent from cGMP-mediated vascular smooth muscle [46]. Overall, a decrease in BP triggered the arterial baroreceptor reflex and increased sympathetic nerve activity (SNA), resulting in tachycardia. Historically, the baroreflex is considered a short-term regulator of BP and HR, with little or no influence chronically. However, our data has challenged this concept, as we observed that long-term treatment with Compound 1 or combination therapy with Enalapril sustained heart rate chronically. Other supportive evidence also confirmed that chronic electrical stimulation of baroreceptor afferents produced sustained decreases in SNA and BP in animal models and in patients with hypertension [47].

Impaired NO-cGMP signaling reduces glomerular blood flow, whereas increasing cGMP increases renal blood flow. The diameter of afferent and efferent capillaries is regulated by blood flow as well as by production of NO and cGMP. While increasing cGMP levels leads to relaxation of both afferent and efferent arterioles, such dilation is more pronounced in efferent arterioles [48]. It was shown that cGMP increases renal blood flow in isolated perfused rat kidneys [49]. Although we did not directly measure cGMP levels during Compound 1 treatment, our data demonstrated that acute IV infusion of Compound 1 in a dose-dependent fashion increased renal blood flow in normal CD rats and ZSF1 obese rats. We postulate that increasing cGMP by administering Compound 1 dilates both the afferent and efferent arterioles, but preferentially dilates the efferent arterioles, in which NO synthases are expressed [50]. Administering Compound 1 leads to increased renal blood flow and chronically enhanced glomerular filtration rates. In combination with Enalapril, Compound 1 further increased renal blood flow acutely and maintained GFR, while in the untreated ZSF1 obese rats DN continued to progress.

Administering Compound 1 alone or in combination with angiotensin-converting enzyme inhibitors (ACEi) significantly enhanced RBF and increased cGMP levels, resulting in vascular relaxation and in reversing hypertension. In a progressive model of DN in ZSF1 obese rats, the context of hypertension increased local pressure in afferent pre-glomeruli capillaries and impaired vascular membrane integrity [51]. That impairment induces proteinuria, which has been demonstrated to be a strong risk factor for renal function decline [52]. Furthermore, reductions in proteinuria with angiotensin II receptor blockers (ARBs) have been shown to be predictive of renal function outcome in patients with DN [52]. The chronic treatment of sGC with Compound 1 alone reduced kidney hypertrophy, significantly slowed the progression of proteinuria, and significantly reduced urinary albumin and creatinine ratio. Indeed, sGC modulators mediated downstream cGMP signaling and exerted a plethoric role in maintaining glomerular filtration function, preserving integrity of kidney structures (tubules, macular densa, and podocytes), and reducing interstitial fibrosis and mesangial cell contraction and expansion [1]. Increasing cGMP levels is predicted as an effective treatment approach to oppose the loss of kidney structure integrity and functionality in diabetic and hypertensive nephropathy in ZSF1 obese rats. However, Compound 1 alone did not have a significant effect on nephropathy scores, while in combination with Enalapril, it significantly decreased the incidence of glomerulosclerosis lesions, as observed by the reduction in interstitial fibrosis and in the tubules, interstitial, and glomeruli nephropathy score. It is likely that the dosage level of Compound 1 was not sufficient to maintain cGMP levels that would normalize BP and reduce renal function, indicating that combination therapies are necessary to attenuate disease progression.

Beyond the hemodynamic renal protective effect, our data suggests that Compound 1 enhanced GFR in combination with Enalapril. After chronic treatment with Compound 1 in ZSF1 obese rats, improvement in renal function was supported by lower levels of plasma creatinine and plasma urea. Circulating levels of Cystatin C were not reduced after Compound 1 treatment. The discordance of plasma Cystatin C levels with other renal function parameters such as plasma creatinine and urea could be influenced by multiple factors other than kidney function, such as cardiovascular disease [53] and C-reactive protein levels [54]. In addition, elimination of Cystatin C via routes other than the kidney has been documented [55]. In theory, Cystatin C has a low molecular weight. If it were only removed from the bloodstream by glomerular filtration in the kidneys, blood levels of Cystatin C would rise and urinary levels of Cystatin C would decrease when kidney function is declining. However, our data demonstrated that, in the presence of Enalapril, with or without Compound 1, lower levels of Cystatin C in plasma resulted in less urinary secretion of Cystatin C. Therefore, our data supports that Cystatin C may be not a superior kidney functional biomarker for cardiorenal disease.

Recent evidence suggests that NGAL and KIM-1 can be released from damaged tubules [56] and both may be involved in the pathophysiological process leading to chronic renal disease, and are correlated with severity of renal impairment. In accordance with global renal functional improvement, NGAL was modestly reduced in the Compound 1 treatment arm. KIM-1 is also known to play a role in renal tubular epithelial cell repair process likely mediated via ERK/MAPK process and inhibit renal inflammatory response [57]. We did not find increases in other renal injury biomarkers other than KIM-1. In addition, compound 1 did not reduce kidney histopathological score in combination therapy compared to enalapril alone. Compound 1 as monotherapy showed significant improvements in GFR, urinary protein to creatinine ratio and urinary albumin to creatinine. The additive effects were observed in combination arm indicating compound 1 improves renal function and not injury. Altogether, it indicates the transient increase in KIM-1 could be more due to renal repair and reduction in inflammation rather than tubular injury. Otherwise, the effects on improvement in kidney score and renal function should have reduced in combination therapy compared to enalapril alone. Further understanding of KIM-1 long term effects needs to be addressed clinically rather than in a six-month pre-clinical study and beyond the scope of this paper.

Our findings indicated that 6 months of treatment with Compound 1, a sGC stimulator, reduced progression of renal damage in the ZSF1 obese rats, thus highlighting the potential of sGC stimulators as effective therapy for DN. Based on the preclinical and RNA-Seq studies, combining an sGC stimulator with standard of care such as Enalapril may provide an additional/synergistic effect on slowing the progression of kidney disease.

RNA-Seq data specifically highlights a superior therapeutic option of combination therapy of the sGC stimulator with Enalapril. The combination therapy activates Farnesoid X receptor/Retinoid X receptor (FXR/RXR), which can regulate the balance of relaxation and contraction in smooth muscle cells. FXR activation protects medullary-collecting duct cells from hypertonicity-induced cell injury [50–58] and exerts anti-inflammatory and antifibrotic effects. Altogether, FXR may be a critical regulatory factor in renal physiology and pathophysiology. It normalizes renal function, thus inhibiting the progression of DN. NRF2 is a key master switch controlling the expression of antioxidant and protective enzymes involved in the regulation of the cellular redox state. It has been reported that diminished activity of NRF2 results in reduced antioxidative potential, apoptosis, and/or necrosis [59]. The relationship between NRF2 activity vs redox reserve capacity and the NO-sGC-cGMP pathway regulated by sGC stimulators may represent one of the key mechanisms underpinning renal protective effects. Further detailed investigation to confirm the beneficial effect of this relationship is warranted. Patient selection guided by endothelial dysfunction and oxidative stress biomarkers might help direct the clinical development of sGC stimulators in combination with standard of care therapies.

## Limitations

The bulk RNAseq provides valuable insight to the sGC mechanism stimulated by compound1. Based on the findings from compound 1 on improving renal endpoints, we think the remodeling & anti-hypertensive effects are from endothelial cells, smooth muscle cells in the renal vasculature. The impact on the glomerulus filtration by compound 1 suggests that it targets both afferent and efferent arterioles. In addition, compound could target both mesangial and fibroblasts based on the histology. The direct effects of compound 1 on different cells in the kidney would require scRNASeq which is a limitation in this study. We did not include riociguat or other sGC stimulators or activators as another control arm. The rationale was riociguat or other sGC compounds on renal effects are reported with different in vivo model(s). In this study another generation of sGC stimulator compound 1 shows renal benefits to reduce renal organ damage either alone or in presence of Enalapril in different in vivo model corroborating with findings reported earlier with riociguat alone. The scope of this study was to highlight that sGC pathway plays an important pathological role in diabetic nephropathy progression and can be therapeutically intervened with different generations of sGC activators or stimulators on top of standard of care for additional renal benefits.

## Supporting information

S1 Fig (TIF)
