# Supplementary figures and images for: Effects of soluble guanylate cyclase stimulator on renal function in ZSF-1 model of diabetic nephropathy
Source: PLoS One. 2022 Jan 27;17(1):e0261000. doi: 10.1371/journal.pone.0261000 (PMC8794189; doi:10.1371/journal.pone.0261000)

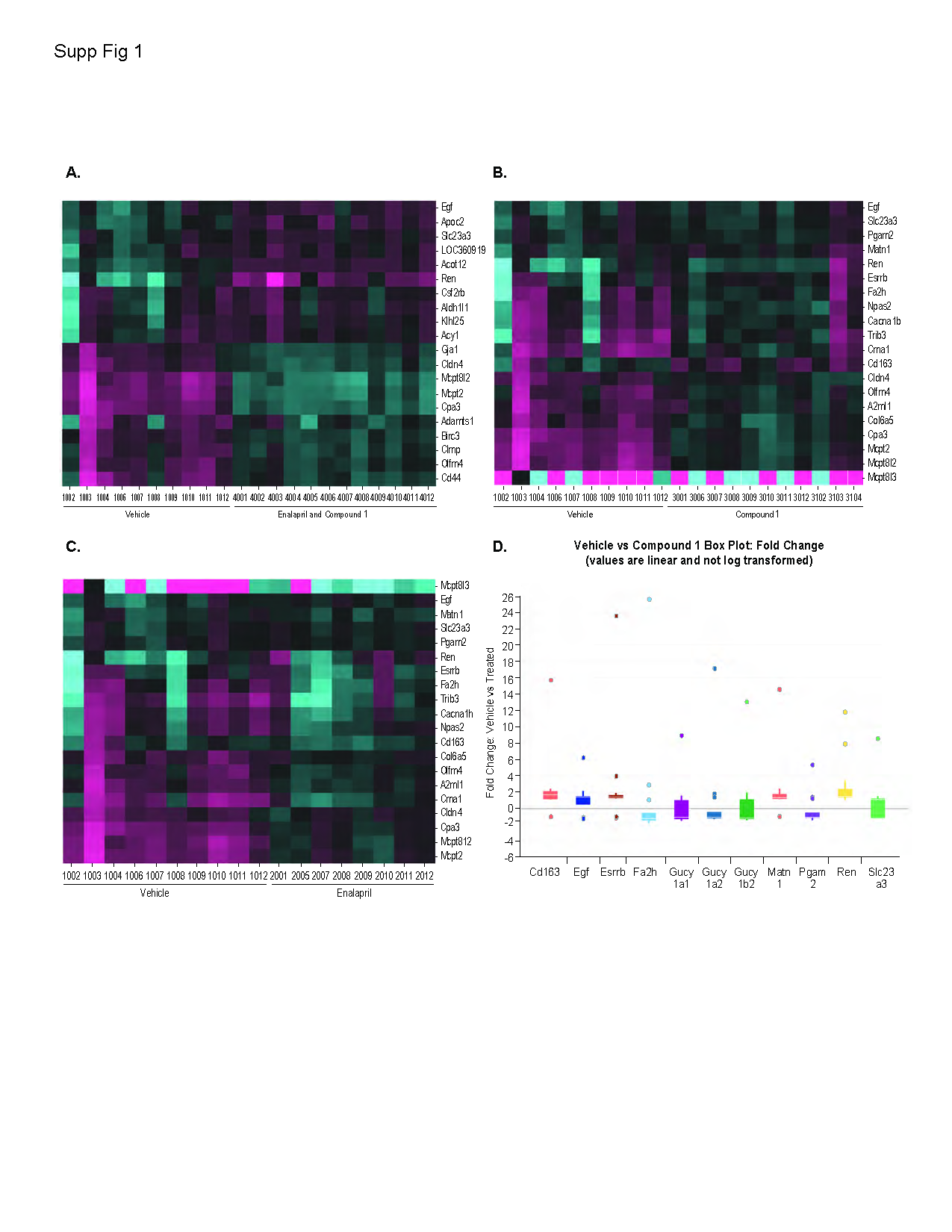

Supplement: S1 Fig — (TIF) [file pone.0261000.s001.tif]
